# Altered somatosensory profiles in rheumatoid arthritis, psoriatic arthritis, axial spondyloarthritis, and systemic sclerosis

**DOI:** 10.1016/j.ero.2025.07.001

**Published:** 2025-08-06

**Authors:** Simon M. Petzinna, Claus-Juergen Bauer, Ruth S. Schrapper, Martin Mücke, Charlotte Behning, Tim T.A. Bender, Pantelis Karakostas, Valentin S. Schäfer

**Affiliations:** 1 Medical Clinic III for Oncology, Hematology, Immune-Oncology and Rheumatology, University Hospital Bonn, Bonn, Germany; 2 Institute for Digitalization and General Medicine, University Hospital of Aachen, Aachen, Germany; 3 Department of Medical Biometry, Informatics and Epidemiology, University Hospital Bonn, Bonn, Germany; 4 Center for Rare Diseases Bonn, University Hospital Bonn, Bonn, Germany

## Abstract

**Objectives:**

This exploratory study aimed to examine the somatosensory profiles of patients with rheumatoid arthritis (RA), psoriatic arthritis (PsA), axial spondyloarthritis (axSpA), and systemic sclerosis (SSc) using quantitative sensory testing (QST). We sought to identify distinct patterns of sensory alterations to enhance the understanding of pain mechanisms in these conditions and to generate hypotheses for future mechanistic research and therapy approaches.

**Methods:**

Patients with RA, PsA, axSpA, and SSc underwent QST on both hands to evaluate all somatosensory submodalities. Standardised assessment, following the German Research Network on Neuropathic Pain protocol, included mechanical detection threshold (MDT) and vibration detection threshold (VDT) as well as thermal detection and pain thresholds.

**Results:**

We enrolled 80 patients (20 with RA, PsA, axSpA, SSc) and 20 controls. Significant differences in MDT (RA: *β* = 0.90, *P* =.025; PsA: *β* = 1.30, *P* =.001; axSpA: *β* = 0.80, *P* =.045; SSc: *β* = 0.86, *P* =.030) and VDT (RA: *β* = −0.33, *P* =.003; PsA: *β* = −0.23, *P* =.033; axSpA: *β* = −0.30, *P* =.006; SSc: *β* = −0.17, *P* =.126) were observed compared with controls. All disease groups exhibited pathological allodynia (RA: 15%, PsA: 25%, axSpA: 15%, SSc: 5%, and controls: 0%), with sensory processing alterations occurring independently of inflamed areas. No association was found between QST-detected sensory alterations and disease activity, duration, or inflammatory markers.

**Conclusions:**

Patients with RA, PsA, axSpA, and SSc demonstrate significant alterations in somatosensory processing, including abnormal MDT and VDT, and pathological allodynia that appear independent of inflamed areas. These sensory changes do not correlate with disease activity, duration, inflammatory markers, or therapeutic approach, indicating that they may result from mechanisms distinct from inflammation.

**WHAT IS ALREADY KNOWN ON THIS TOPIC**

• Chronic pain is a frequent symptom in patients with rheumatologic diseases such as rheumatoid arthritis (RA), psoriatic arthritis (PsA), axial spondyloarthritis (axSpA), and systemic sclerosis (SSc), persisting even under effective anti-inflammatory treatment.
• Pain in these diseases is not solely driven by active inflammation but may also involve central sensitization and dysregulation of pain processing pathways.
• Quantitative sensory testing (QST) is a standardized method for assessing somatosensory function and has been utilized to identify alterations in pain perception, primarily in RA and axSpA.

**WHAT THIS STUDY ADDS**

• This is the first study to systematically assess somatosensory profiles using a standardized QST protocol across four rheumatologic diseases (RA, PsA, axSpA, and SSc) in comparison to healthy controls.
• Patients with all four conditions demonstrated increased mechanical detection thresholds (MDT) and decreased vibration detection thresholds (VDT), suggesting widespread Aβ-fiber dysfunction.
• Pathological allodynia was observed across all patient groups, independent of inflammation, disease activity, or treatment regimen.
• Distinct thermal profiles, such as heightened cold sensitivity in SSc and delayed warm detection in RA, indicate disease-specific involvement of Aδ and C fibers.

**HOW THIS STUDY MIGHT AFFECT RESEARCH, PRACTICE OR POLICY**

• The findings underscore the need to expand pain assessment in rheumatology beyond inflammatory markers, integrating mechanism-based diagnostics such as QST.
• Somatosensory profiling may guide individualized pain management strategies by identifying central or peripheral sensitization as therapeutic targets.
• Future clinical trials may benefit from stratifying patients by sensory phenotype to improve treatment response and outcome evaluation.

Alt-text: Unlabelled box

## INTRODUCTION

Pain is a predominant symptom across various rheumatologic diseases, including rheumatoid arthritis (RA), psoriatic arthritis (PsA), axial spondyloarthritis (axSpA), and systemic sclerosis (SSc). Despite notable pain alleviation achieved through the efficacy of disease-modifying antirheumatic drugs (DMARDs) in targeting peripheral inflammation, residual pain may persist even in patients achieving disease remission [[1], [2], [3]]. This chronic pain can significantly impact overall well-being, resulting in sleep disturbances, functional disability, and psychosocial distress [4,5]. However, there exists a lack of awareness among physicians, as studies indicate a disparity between the activity and impact of disease as assessed by physicians compared with patient-reported pain [6,7].

The intensity, distribution pattern, and nature of pain in rheumatologic diseases are influenced by multiple factors [1,8]. Previous research underscores the role of both peripheral inflammation and sensitisation, as well as dysregulation in the central nervous system, in pain manifestation within rheumatologic conditions [[8], [9], [10]]. Particularly, central pain regulatory mechanisms (including descending facilitatory and inhibitory pathways) and central sensitisation emerge as pivotal in the transition from acute to chronic pain [11,12]. The descending facilitatory and inhibitory pathways originate in the brain, passing through the brainstem to the spinal cord [1]. A critical structure involved is the periaqueductal gray, which receives inputs from the frontal cortex, amygdala, and hypothalamus, pertaining to factors such as stress and mood that influence pain perception [13]. Information is further integrated and relayed to the brainstem and the rostral ventromedial medulla. The rostral ventromedial medulla plays a crucial role in either suppressing or facilitating pain, depending on the pathways activated [1]. Eventually, central sensitisation occurs in the dorsal horn of the spinal cord and is characterised by increased neural excitability, enhanced excitatory input, reduced inhibitory activity, and heightened pain sensitivity [1]. It encompasses an acute phase, driven by N-methyl-D-aspartate receptor activation by glutamate, and a chronic phase, involving the transcription of pain-regulating peptides and the activation of spinal microglia [1,14]. In RA and axSpA, central sensitisation has been found to cause an increase in central nervous system pain sensitivity, yielding clinical symptoms such as hyperalgesia and allodynia [10,12].

Inflammatory cytokines also appear to play a significant role in both central and peripheral sensitisation, as receptors for tumour necrosis factor-α, interleukin-1β, and interleukin-17 have been detected on sensory neurons, directly influencing the responses of nociceptive neurons [[15], [16], [17], [18]]. Consequently, despite the absence of apparent permanent tissue damage, a prolonged elevation in the sensitivity of afferent nociceptive fibres can be observed [19,20]. Additionally, small-fibre neuropathy has been reported as a potential cause of persistent peripheral pain [21,22].

Despite its clinical significance, the assessment of peripheral and central sensitisation in humans remains challenging. One promising diagnostic tool is quantitative sensory testing (QST). By applying calibrated thermal and mechanical stimuli in a standardised approach, QST facilitates the evaluation of various nerve fibre groups, identifying specific sensory abnormalities such as hyperalgesia or hypoesthesia, indicative of underlying pathologies [[23], [24], [25], [26]]. Data on QST in rheumatologic diseases are currently limited, with most studies predominantly focusing on RA and axSpA [12,21,27]. These studies have revealed decreased pressure pain thresholds (PPT) observed both in inflamed and in noninflamed areas, indicating a generalised hyperalgesia beyond localised inflamed joints.

Our study aimed to explore somatosensory profiles in patients with RA, PsA, axSpA, and SSc utilising QST. We aimed to characterise patients’ responses to mechanical and thermal stimuli and identify patterns of sensory alteration, thereby shedding light on the underlying mechanisms of pain in these conditions.

## METHODS

### Patient characteristics

Patients diagnosed with RA, PsA, axSpA, and SSc were prospectively enrolled in the study between July 1, 2022, and November 30, 2022. Recruitment occurred directly through the Department of Rheumatology and Clinical Immunology at the University Hospital of Bonn, Germany. Diagnosis was confirmed by a board-certified rheumatologist, and patients were required to meet the respective classification criteria: the American College of Rheumatology (ACR)/European League Against Rheumatism (EULAR) 2010 criteria for RA [28], the Classification for Psoriatic Arthritis (CASPAR) Criteria for PsA [29], the Assessment of SpondyloArthritis International Society (ASAS) 2009 criteria for axSpA [30,31], and the ACR/EULAR 2013 criteria for SSc [32]. Furthermore, patients were required to have the physical and mental capacity to participate in the study, be aged between 18 and 65 years, and provide written informed consent. Exclusion criteria included pregnancy, nursing period, or skin and soft tissue diseases. Control patients, matched for age, gender, and disease history, were also recruited from the same department and were mandated to have no history of rheumatologic conditions.

For each patient, demographic data, disease history, medication, current symptoms, disease activity scores, and current pain levels were documented. The disease activity scores used were the ‘Clinical Disease Activity Index’ (CDAI) for RA, the ‘Disease Activity in Psoriatic Arthritis’ (DAPSA) and ‘Psoriatic Arthritis Disease Activity Score’ (PASDAS) for PsA, the ‘Ankylosing Spondylitis Disease Activity Score’ (ASDAS) and ‘Bath Ankylosing Spondylitis Disease Activity Index’ (BASDAI) for axSpA, and the ‘modified Rodnan Skin Score’ (mRSS) for SSc. Routine laboratory tests, including C-reactive protein (CRP) measurements, were performed.

### QST

**Figure 1:**
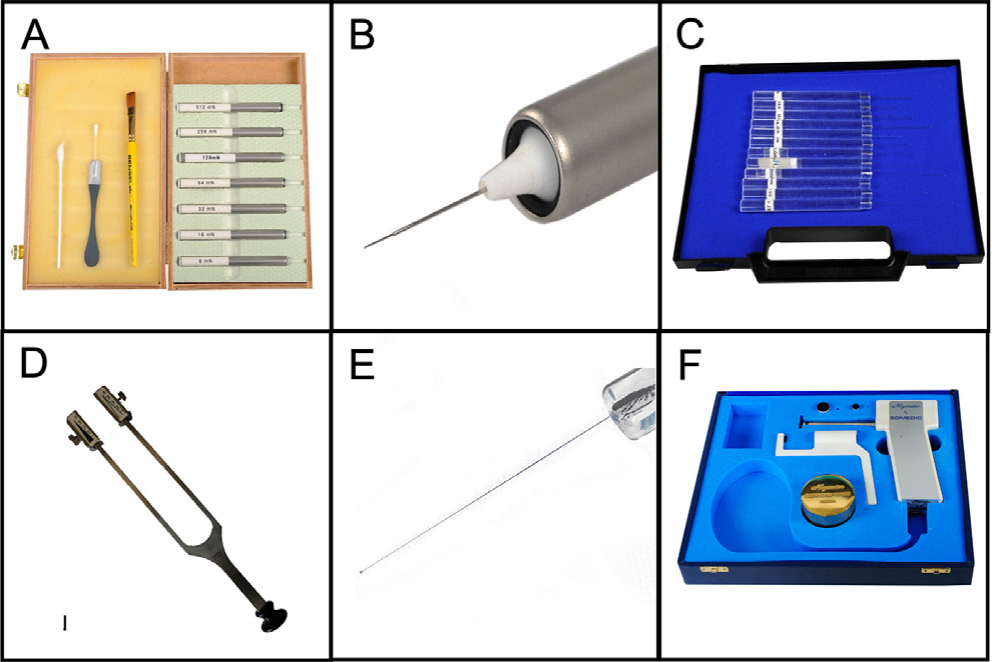
Mechanical testing set. These tools allow for a comprehensive evaluation of the mechanical aspects of sensory perception and pain sensitivity, facilitating a nuanced understanding of the somatosensory system’s functionality [26]. A, Pinprick stimulators of varying intensity, as well as cotton swabs, cotton balls, and brushes. B, A pinprick stimulator. C, von Frey filaments for checking the mechanical detection threshold. D, Filaments made of glass fibre cable with a rounded tip. E, A neurologic 64-Hz tuning fork with an 8/8 scale (Rydel-Seiffer) for checking the vibration threshold. F, A digital pressure algometer for determining the pressure pain threshold.

**Figure 2:**
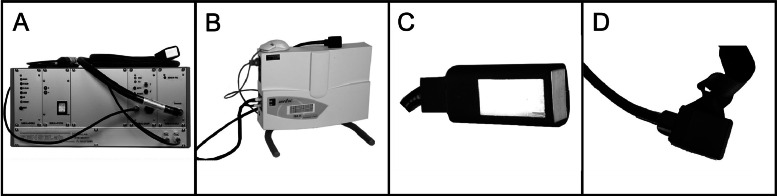
Thermal testing set. Thermal testing involves the use of equipment such as a modular sensory analyser (A) and a thermal sensory analyser (B). Water-perfused thermodes (C, D) are fixed onto the testing area with the Peltier element side, which, depending on the control, leads to a cooling or warming of the skin. The thermotester is connected to a computer via an interface, which controls the device and records the threshold values found [26].

Patients with RA, PsA, axSpA, and SSc, as well as control patients, underwent standardised QST on both hands following the German Research Network on Neuropathic Pain (DFNS) protocol [[24], [25], [26]]. The procedure, carried out once by a certified examiner (RSS), included calibrated mechanical (Fig 1) and thermal (Fig 2) test stimuli compiled into 7 tests measuring a total of 13 parameters (Table 1). These tests were categorised into 4 groups: thermal detection thresholds, thermal pain thresholds, mechanical detection thresholds (MDTs), and mechanical pain thresholds (MPTs).

**Table 1:** Sensory stimuli mediation and associated nerve fibre types

Small fibre–mediated stimuli	
Cold detection threshold (CDT)	A δ-Fibres
Warm detection threshold (WDT)	C-Fibres
Thermal sensory limen (TSL)	A δ-Fibres, C-Fibres
Paradoxical heat sensations (PHS)	A δ-Fibres, C-Fibres
Cold pain threshold (CPT)	A δ-Fibres, C-Fibres
Heat pain threshold (HPT)	A δ-Fibres, C-Fibres
Mechanical pain threshold (MPT)	A δ-Fibres, C-Fibres
Mechanical pain sensitivity(MPS)	A δ-Fibres, C-Fibres
Wind-up ratio (WUR)	A δ-Fibres, C-Fibres
Pressure pain threshold (PPT)	A δ-Fibres, C-Fibres
Allodynia	A δ-Fibres, C-Fibres

Large fibre–mediated stimuli	

Vibration detection threshold (VDT)	Aβ-Fibres
Mechanical detection threshold (MDT)	Aβ-Fibres
Allodynia (ALL)	Aβ-Fibres

The table displays the thermal and mechanical test stimuli tested in the quantitative sensory testing protocol and their associated nerve fibre types. Findings include differentiation of stimuli mediated by small fibres (Aδ-Fibres, C-Fibres) and large fibres (Aβ-Fibres).

### MDTs

MDTs were measured using a set of standardised von Frey filaments (OptiHair2-Set, Marstock Nervtest). The filaments are made of glass fibre and vary in diameter and length, featuring a spherical contact area that measures approximately 0.5 mm in diameter. For accurate testing, each filament was applied until it bent into an S-shape. The test set included filaments of strengths ranging from 0.25 to 512 mN. During testing, each filament’s contact time with the skin surface was limited to 2 seconds. The tactile detection threshold was determined by calculating the geometric mean of 5 just supra- and subthreshold stimulus intensities, using a modified Levels method. The MDT was assessed by averaging the ascending and descending stimulus intensities between 0.25 and 512 mN. Apart from that, for the vibration detection threshold (VDT) assessment, a 64-Hz tuning fork (Ryder-Seiffer graded) with an 8/8 scale was placed on the processus styloideus ulnae.

### Mechanical pain thresholds

To assess MPT, mechanical pain sensitivity (MPS), wind-up ratio (WUR), and allodynia, we employed weighted pinprick stimulators with forces ranging from 8 to 512 mN (Pinprick, MRC Systems GmbH). The MPT was determined using the method of limits for ascending and descending stimuli, pinpointing the threshold at which a stimulus transitions from being perceived as sharp to blunt. MPS and allodynia were evaluated using a fixed series of pinprick stimuli alongside nonpainful stimuli (such as a brush, cotton ball, or cotton wool tip stimuli). The WUR was calculated by comparing the pain perception of a single stimulus against a series of 10 stimuli of identical intensity. Additionally, the PPT was measured using an algometer device (Somedic), which applies increasing force to the thenar muscles.

### Thermal detection thresholds

The cold detection threshold (CDT) and warm detection threshold (WDT), as well as the thermal sensory limen (TSL), were assessed using the neurosensory testing device (TSA II-2001, Medoc). The thermodes used in the device had a contact area of 9.0 cm². Administration of alternating warm and cold stimuli, starting from the baseline temperature of 32°C, was performed to assess paradoxical heat sensations (PHSs). The temperature change rate of the device was set at 1°C/s. The device was programmed to automatically shut off upon reaching temperatures of either 0°C or 50°C, subsequently returning to the initial temperature of 32°C.

### Thermal pain thresholds

For assessing the cold pain detection threshold (CPT) and warm pain detection threshold (WPT), participants were instructed to stop a decreasing or increasing temperature stimulus when they experienced burning or prickling sensations. This threshold determination was executed by the participants themselves, who were provided with a stop switch.

### Statistical analysis

The QST data, with the exception of CPT, heat pain threshold (HPT), VDT, and PHS, was normalised using a log10 transformation [33]. To prevent the loss of data points during this transformation, a fixed value of 0.1 was added to each value to avoid recording zeroes, as described before [34]. The analysis then proceeded with linear regression models, where the somatosensory profile was treated as the dependent variable and the diagnostic category as the independent variable. An adjustment was made for each patient’s CRP concentration in serum to account for potential confounding effects.

For comparison purposes, individual somatosensory profiles were evaluated against a normative dataset from a healthy control group. This was done to identify any diagnosis-specific somatosensory abnormalities. A Z-transformation was applied to this comparison for ease of interpretation. In this scheme, positive Z-scores indicated increased sensitivity, whereas negative scores pointed to reduced sensitivity within the somatosensory parameters. Values falling outside the 95% CI were considered to represent pathological deviations. The presence of allodynia and PHS was inherently considered pathological, as described before [[24], [25], [26]].

### Ethical approval

The study was conducted in accordance with the Declaration of Helsinki and has been reviewed and approved by the ethics committee of the University Hospital Bonn, Germany (reference number: 065/20). Written informed consent was obtained from every patient prior to inclusion in the study.

## RESULTS

### Patient characteristics

In this study, we prospectively enrolled 80 patients, with 20 patients each having RA, PsA, axSpA, and SSc. Additionally, a control group of 20 patients, matched for age and gender, was included in the study. Demographics, patient characteristics, and disease-specific as well as analgesic medication at the time of inclusion are depicted in Table 2. Regarding disease activity assessment in RA patients, the mean CDAI was recorded at 11.9, with an SD of ±10.4. In the PsA subgroup, the mean DAPSA score was 20.3 (SD ±11.0), whereas the PASDAS averaged at 3.7 (SD ±1.6). For axSpA patients, the mean ASDAS was 1.8 (SD ±0.8), and the BASDAI yielded a mean score of 2.1 (SD ±1.6). Lastly, in the SSc cohort, the mean mRSS was 9.5 (SD ±6.7), and the ACR/EULAR composite score averaged at 14.75 (SD ±4.1).

**Table 2:** Demographic and clinical data

		Rheumatoid arthritis	Psoriatic arthritis	Axial spondyloarthritis	Systemic sclerosis	Control patients
Patient characteristics		(*n* = 20)	(*n* = 20)	(*n* = 20)	(*n* = 20)	(*n* = 20)
Sex	Male	12 (60%)	7 (35%)	13 (65%)	5 (25%)	10 (50%)
	Female	8 (40%)	13 (65%)	7 (35%)	15 (75%)	10 (50%)
Age (y)	Mean SD	54 (±8.6)	50 (±10.1)	42 (±11.9)	55 (±9.8)	47 (±15.2)
Body mass index (kg/m^2^)	Mean SD	27.3 (±3.8)	28.5 (±4.7)	27.3 (±6.4)	23.3 (±4.2)	24 (±3.2)
C-reactive protein (mg/L)	Mean SD	5.9 (±11.8)	2.6 (±4.3)	2.4 (±4.1)	1.8 (±3.3)	N/A
Prednisolone	*N* (%)	2 (10%)	3 (15%)	2 (10%)	0 (0%)	0 (0%)
csDMARD	*N* (%)	12 (60%)	11 (55%)	0 (0%)	12 (60%)	0 (0%)
bDMARD	*N* (%)	7 (35%)	7 (35%)	14 (70%)	0 (0%)	0 (0%)
tsDMARD	*N* (%)	4 (20%)	4 (20%)	3 (15%)	0 (0%)	0 (0%)
NSAID	*N* (%)	0 (0%)	1 (5%)	7 (35%)	1 (5%)	0 (0%)

bDMARD, biological disease-modifying antirheumatic drug; csDMARD, conventional synthetic disease-modifying antirheumatic drug; NSAID, nonsteroidal anti-inflammatory drug; tsDMARD, targeted synthetic disease-modifying antirheumatic drug.

Table 2 presents the epidemiological characteristics of the study population, according to their rheumatologic diagnosis.

### QST

**Figure 3:**
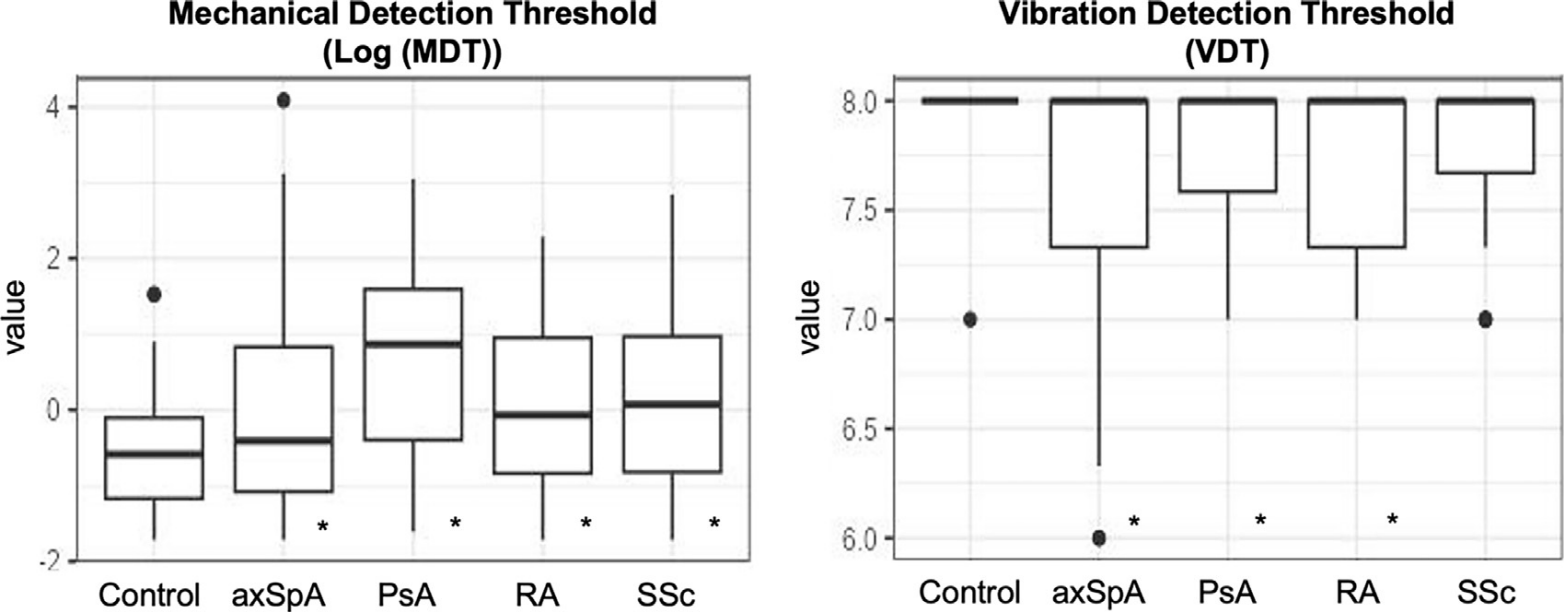
Mechanical detection and vibration detection thresholds in rheumatoid arthritis, psoriatic arthritis, axial spondyloarthritis and systemic sclerosis patients. This figure compares the mechanical detection threshold (MDT) and vibration detection threshold (VDT) in rheumatoid arthritis, psoriatic arthritis, axial spondyloarthritis, and systemic sclerosis patients to control group patients. The MDT and VDT are depicted as logarithmic values and direct measurements, respectively. Statistical significance is indicated by *P* values, with *P* < .05 considered significant.

QST examinations of MDT revealed significant abnormalities across various rheumatologic conditions. Specifically, patients with RA (*β* = 0.90, 95% CI: 0.12-1.68, *P* = .025), PsA (*β* = 1.30, 95% CI: 0.52-2.09, *P* = .001), axSpA (*β* = 0.80, 95% CI: 0.02-1.58, *P* = .045), and SSc (*β* = 0.86, 95% CI: 0.08-2.65, *P* = .030) displayed significantly higher MDT values than control patients. Additionally, a significant reduction in VDT was observed in patients with RA (*β* = −0.33, 95% CI: −0.55 to −0.12, *P* = .003), PsA (*β* = −0.23, 95% CI: −0.45 to −0.02, *P* = .033), and axSpA (*β* = −0.30, 95% CI: −0.51 to −0.09, *P* = .006) (Table 3, Fig 3). Although patients with SSc exhibited a trend towards reduced VDT, this finding lacked statistical significance (*P* = .126).

**Table 3:** Analysis of mechanical detection and vibration detection thresholds in patients with rheumatoid arthritis, psoriatic arthritis, axial spondyloarthritis, and systemic sclerosis

	Mechanical detection threshold (Log [MDT])			Vibration detection threshold (VDT)		
Patient characteristics	Estimates (*ß*)	95% CI	*P* value	Estimates (*ß*)	95% CI	*P* value
Rheumatoid arthritis	0.90	0.12-1.68	.025*	−0.33	−0.55 to −0.12	.003*
Psoriatic arthritis	1.30	0.52-2.09	.001*	−0.23	−0.45 to −0.02	.033*
Axial spondyloarthritis	0.80	0.02-1.58	.045*	−0.30	−0.51 to −0.09	.006*
Systemic sclerosis	0.86	0.08-1.65	.030*	−0.17	−0.38 to 0.05	.126

This table displays the estimates (ß), 95% CI, and *P* values for mechanical detection thresholds (MDT) and vibration detection threshold (VDT) across rheumatoid arthritis, psoriatic arthritis, axial spondyloarthritis, and systemic sclerosis patients compared with control group patients. The MDT and VDT are recorded as logarithmic values and direct measurements, respectively. Statistical significance is indicated by *P* values, with *P* < .05 considered significant and is marked bold with asterisks..

Linked to MPT, all patient groups showed a notable prevalence of allodynia: specifically, 15% of patients with RA, 25% of patients with PsA, 15% of axSpA, and 5% of patients with SSc, but no control group patients exhibited allodynia. However, other parameters such as MPT, MPS, and WUR did not show significant deviations from the control group in our linear model.

In terms of thermal detection and pain thresholds, SSc patients demonstrated a premature perception of cold as pain (CPT: *β* = 7.33, 95% CI: 1.63-13.02, *P* = .012) but had a delayed response to cold detection (CDT: *β* = −0.46, 95% CI: −0.86 to −0.07, *P* = .023). Patients with RA showed a significantly delayed perception of warm stimuli (WDT: *β* = 0.35, 95% CI: 0.00-0.69, *P* = .004), whereas patients with PsA and axSpA displayed a similar but nonsignificant trend (PsA: *P* =.065, axSpA: *P* =.108). No significant differences were observed in WPT, TSL, and PHS among patients with RA, PsA, axSpA, or SSc compared with the control group. The detailed QST results are depicted in Table 1. Within all patient groups with rheumatologic conditions, no significant association between QST results and CRP concentrations, disease activity scores, or current medication could be shown (data not shown).

## DISCUSSION

The primary objective of our study was to investigate somatosensory profiles utilising QST in patients with RA, PsA, axSpA, and SSc in comparison with a healthy control group. We aimed to assess their response to mechanical and thermal stimuli, with the goal of contributing to a deeper understanding of the complex nature of pain experienced in these rheumatologic diseases.

QST has attracted increasing interest as a potential diagnostic tool in recent years. It enables the generation of a comprehensive and standardised somatosensory profile, encompassing both positive and negative sensory signs [[24], [25], [26]]. The protocol established by the DFNS facilitates the assessment of nociceptive and nonnociceptive submodalities in afferent pathways of Aδ-Fibres, Aβ-Fibres, and C-Fibres [24]. Mechanism-based pain diagnostics using QST hypothesise that changes in perception and pain thresholds may indirectly reflect specific neurobiological mechanisms [35].

In our study, we observed a significant increase in MDT and a decrease in VDT across all 4 disease entities (RA, PsA, axSpA, and SSc) compared with the healthy control group. These changes, particularly mediated by Aβ-fibres [25,35,36], suggest a common phenotype in patients with these conditions. Notably, there was no evident association between these sensory alterations and disease activity or duration, contradicting previous reports, which showed that high pain sensitisation is associated with elevations in disease activity measures [5].

Regarding thermal detection and pain thresholds, patients with SSc exhibited increased sensitivity to cold, with a premature perception as pain, but showed a delayed response to cold stimuli. Patients with RA had a significantly slower response to warm stimuli, a trend also observed, though not significantly, in patients with PsA and axSpA. These findings implicate the involvement of thinly myelinated Aδ fibres and unmyelinated C-Fibres in these conditions [25,26]. The heightened sensitivity to cold stimuli in patients with SSc might be attributable to the co-occurrence of Raynaud syndrome. The reasons behind RA patients’ delayed warm stimulus perception remain speculative, potentially involving possible interactions between central nervous processing and the perception mechanisms mediated by Aδ and C-Fibres, as well as frequent heat application for self-treatment.

Additionally, all patient groups displayed a notable prevalence of allodynia, which is considered pathological, as it is generally not present in healthy individuals [[24], [25], [26]]. The occurrence of allodynia at extra-articular sites suggests potential widespread hyperalgesia in these patients, as it has already been shown in the context of juvenile arthritis [27].

Our observations align with generalised alterations in mechanical, vibration, and thermal stimuli modalities beyond localised inflammation areas, indicating global changes in peripheral and central pain processing pathways. Persistent inflammation may trigger the release of proinflammatory cytokines, prostaglandins, peptides, and growth factors from affected tissues, thereby contributing not only to peripheral but also central sensitisation [37]. Therefore, QST and related methodologies, beyond their research utility, hold promise for individualised pain phenotyping and mechanism-based clinical decision-making. In patients with refractory pain, QST may help identify central sensitisation as a therapeutic target. In clinical trials, it can facilitate the stratification of pain phenotypes based on underlying mechanisms and, in selected cases, inform pharmacologic and rehabilitative treatment adaptations according to somatosensory profiles. Accordingly, pain management strategies should extend beyond anti-inflammatory therapy to include mechanism-based approaches. Pharmacologic options, such as anticonvulsants, antidepressants, or other agents targeting neuropathic pain, may be appropriate for addressing pain hypersensitivity. Nonpharmacologic interventions, including cognitive-behavioural therapy, physical activity, and lifestyle modification, can activate endogenous pain-inhibitory pathways [38,39] and have demonstrated efficacy in rheumatic diseases [40].

For a more comprehensive pain assessment, QST may be complemented by patient-centred tools such as body pain diagrams [41] and discordance scores [42], which capture spatial and subjective dimensions of pain. These tools may be particularly relevant in later disease stages, depicting long-term alterations in central pain processing. Thus, chronic disease may lead to cumulative neuroplastic changes and maladaptive coping strategies, perpetuating pain independently of inflammatory activity [[8], [9], [10]].

In this context, pain in autoimmune rheumatologic diseases frequently co-occurs with fatigue, mood disturbances, and reduced well-being, reflecting shared biological pathways such as inflammation, stress axis dysregulation, and central nervous system changes [4,5]. These symptoms may further shape illness perception and cognitive-affective processes, such as anxiety, catastrophising, or negative expectations, which, in turn, modulate pain experience and reporting [6,7]. Although these domains were not assessed in the present study, future research should incorporate them to enable integrated multisymptom profiling and guide multimodal therapeutic strategies. Ultimately, a multidimensional assessment of pain may improve mechanistic understanding and support individualised, mechanism-based treatment planning.

In summary, this research represents a pioneering effort in examining somatosensory profiles of patients with RA, PsA, axSpA, and SSc using QST. Our findings reveal significant alterations in sensory processing in all 4 conditions compared with healthy controls. Specifically, patients exhibited increased MDT and reduced VDT, indicating a common phenotype characterised by impaired Aβ-fibre function. Additionally, we observed pathological allodynia across all patient groups, highlighting the prevalence of abnormal pain sensitivity beyond inflamed areas. Notably, these sensory alterations appeared independent of disease activity, duration, or inflammatory markers, suggesting that persistent pain in these conditions is likely driven by central and peripheral sensitisation mechanisms rather than active inflammation alone. The unique thermal detection profiles, particularly in patients with SSc who exhibited heightened sensitivity to cold, further underscore the complex nature of pain in these rheumatologic diseases. The use of QST, as demonstrated, provides valuable insights into the sensory alterations in RA, PsA, axSpA, and SSc, paving the way for more personalised and effective pain management approaches in clinical practice.

Although our findings contribute to a deeper understanding of the mechanisms underlying chronic pain, they should be interpreted within the exploratory nature of the study, and some limitations must be acknowledged. Despite rigorous standardisation efforts, QST remains dependent on consistent implementation and patient cooperation. Individual factors, such as environmental and psychological conditions, influence its objectivity. Moreover, although individual test results were compared with normative data derived from multicentric studies, one must consider that most QST parameters significantly vary depending on the specific body region and tissue examined, as well as the age of the individual and the current anti-inflammatory and analgesic treatment regime. Although no association was observed between therapy and QST outcomes, the heterogeneity of treatment regimens and the modest sample size preclude definitive conclusions regarding treatment effects. The potential benefits of expanding test areas to include regions affected by inflammation, thereby possibly enhancing diagnostic accuracy, remain uncertain. Future research should aim to include larger and more stratified cohorts to better investigate treatment-related influences on somatosensory phenotypes and to track longitudinal changes in newly diagnosed patients. This would also allow a deeper analysis of disease stage–related differences in pain processing and sensitisation, which have not been comprehensively assessed in our study. Additionally, further research is needed to elucidate the mechanisms underlying pain hypersensitivity in these conditions.

## Competing interests

The authors report no disclosures relevant to the manuscript.
